# Supplementary material for: A supergene underlies linked variation in color and morphology in a Holarctic songbird
Source: Nat Commun. 2021 Nov 25;12:6833. doi: 10.1038/s41467-021-27173-z (PMC8616904; doi:10.1038/s41467-021-27173-z)
Supplement: Supplementary file 1 — Supplementary Information [file 41467_2021_27173_MOESM1_ESM.pdf]

A supergene underlies linked variation in color and morphology in a Holarctic songbird

Erik R. Funk, Nicholas A. Mason, Snæbjörn Pálsson, Tomáš Albrecht, Jeff Johnson, Scott A. Taylor

SUPPLEMENTARY INFORMATION

Supplementary Figure 1

Supplementary Figure 2

Supplementary Figure 3

Supplementary Figure 4

Supplementary Figure 5

Supplementary Figure 6

Supplementary Table 1

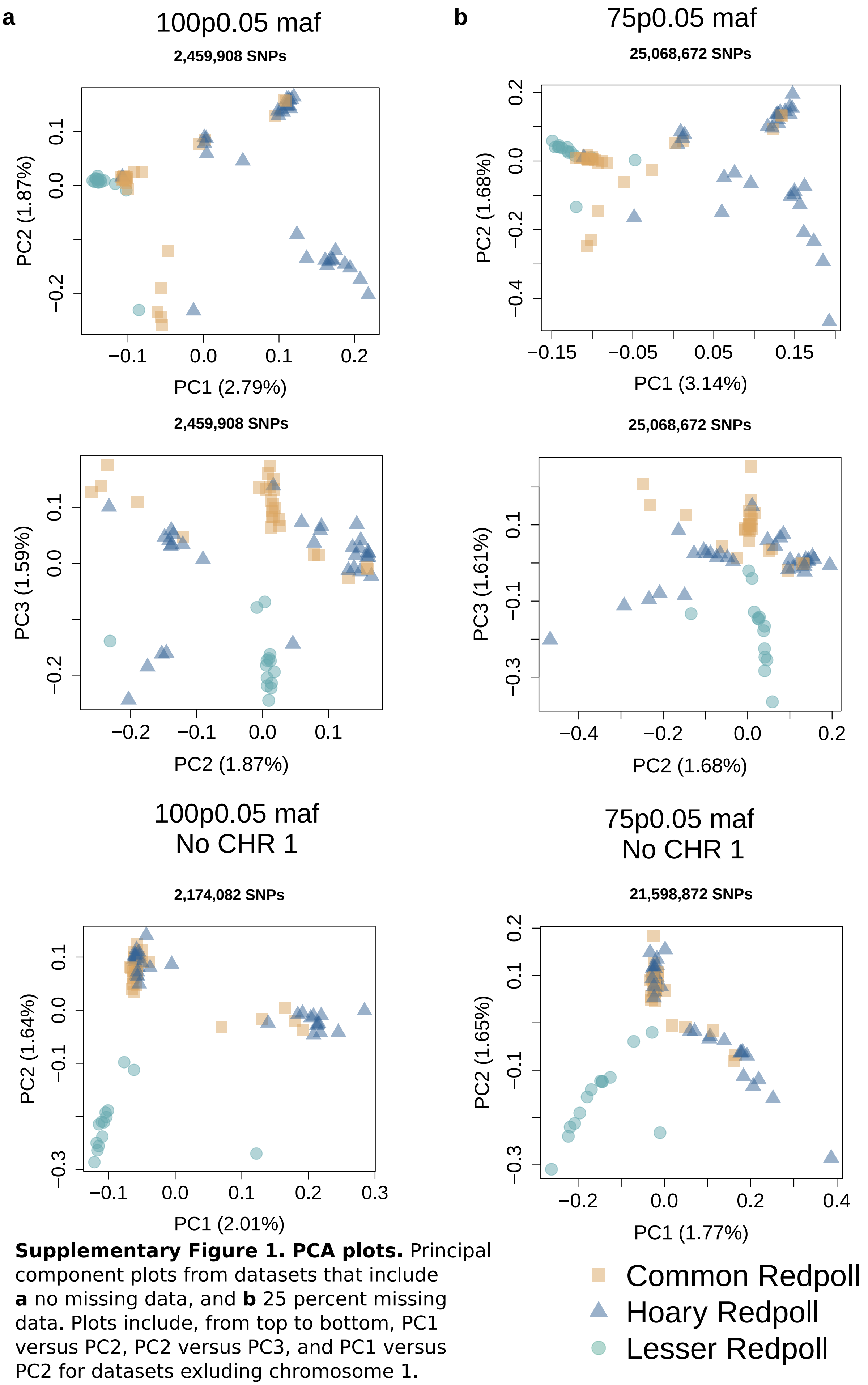

Supplementary Figure 1. PCA plots. Principal component plots from datasets that include **a** no missing data, and **b** 25 percent missing data. Plots include, from top to bottom, PC1 versus PC2, PC2 versus PC3, and PC1 versus PC2 for datasets exluding chromosome 1. Source data are provided as Source Data file.

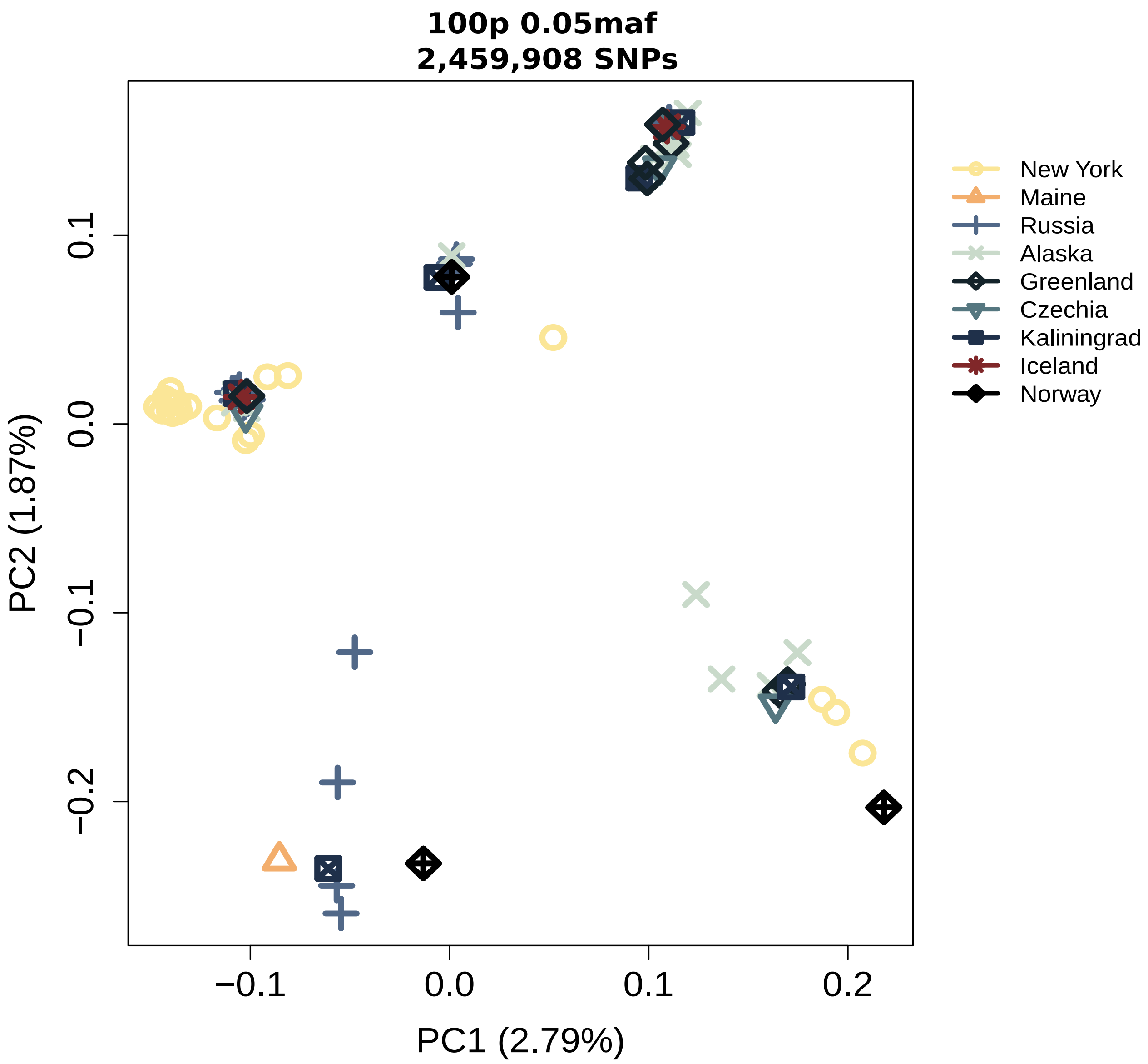

Supplementary Figure 2. PCA by locality. Principal component plot from the dataset that includes SNPs with no missing data, with individuals coded by broad locality. Source data are provided as Source Data file.

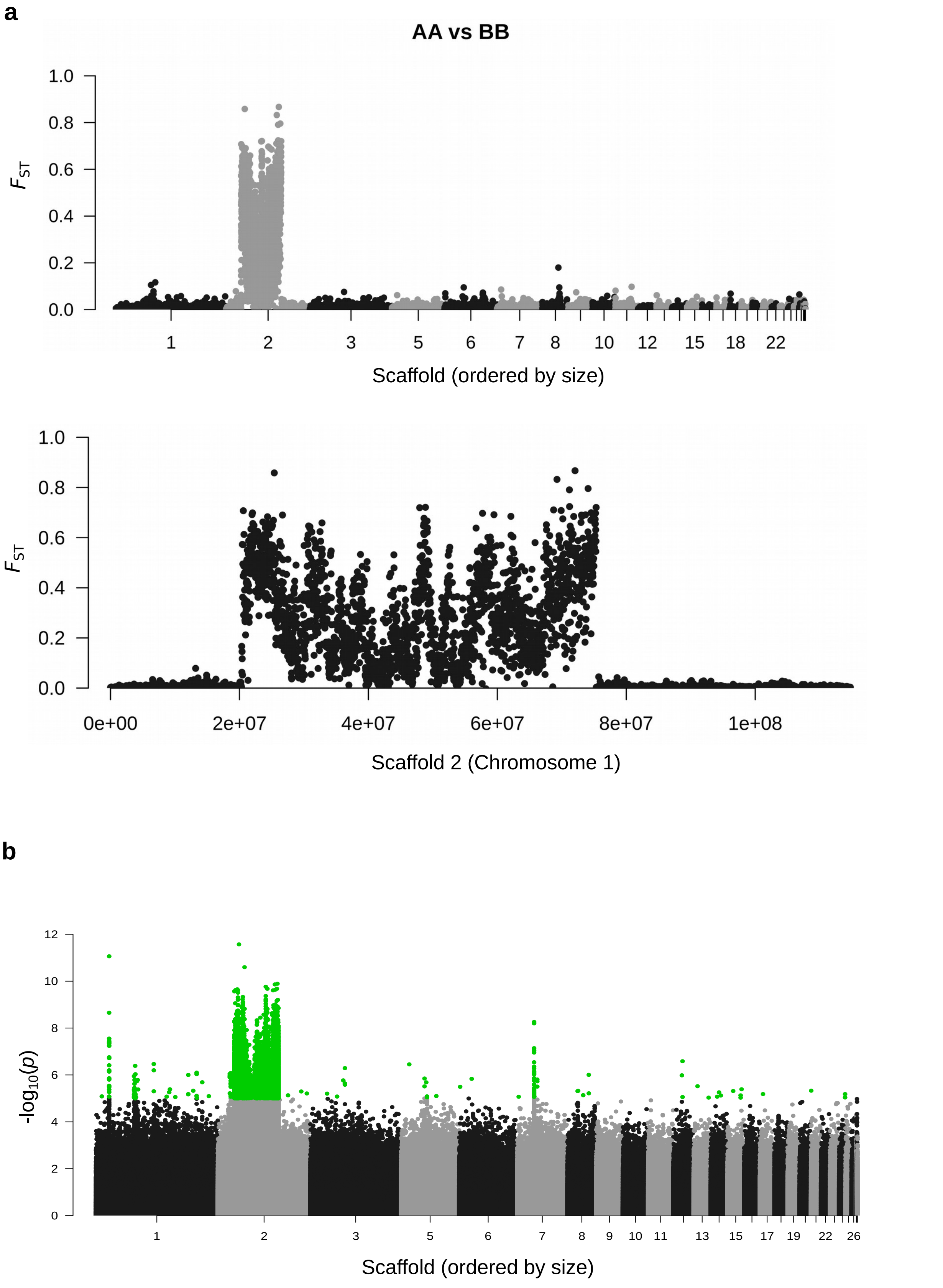

Supplementary Figure 3. Genome-wide manhattan plots. a Pairwise F_{ST} between the AA and BB inversion genotype groups, defined by PCA, and **b** SNPs significantly associated with phenotype using mixed model analysis in GEMMA with an alpha of $1x10^{-5}$ to correct for multiple comparisons.

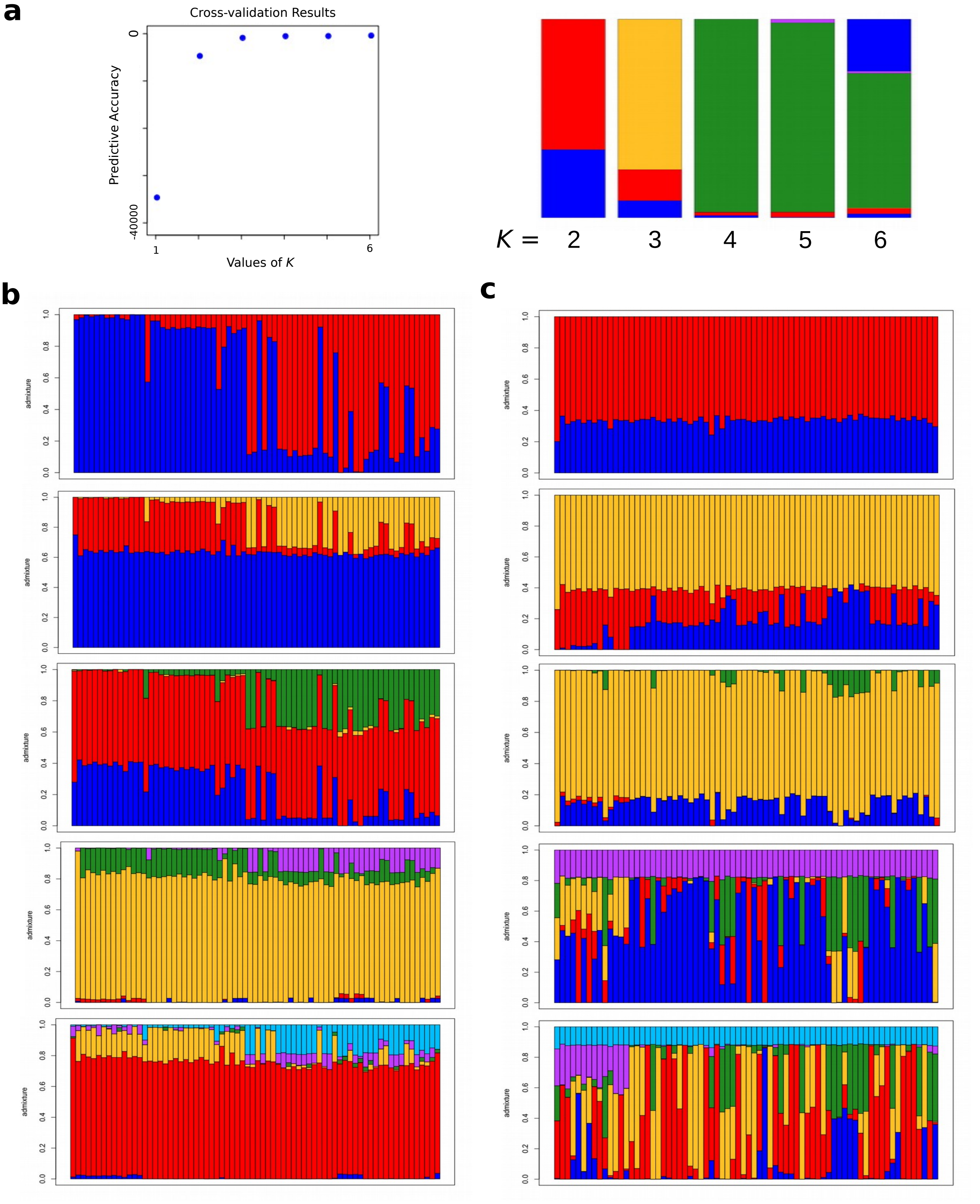

Supplementary Figure 4. ConStruct admixture plots. a Cross validation and layer contributions for values of *K* from 2 to 6. **b** Plots of ancestry proportions for 1 million randomly selected SNPs. **c** Plots of ancestry proportions for 1 million randomly selected SNPs excluding chromosome 1.

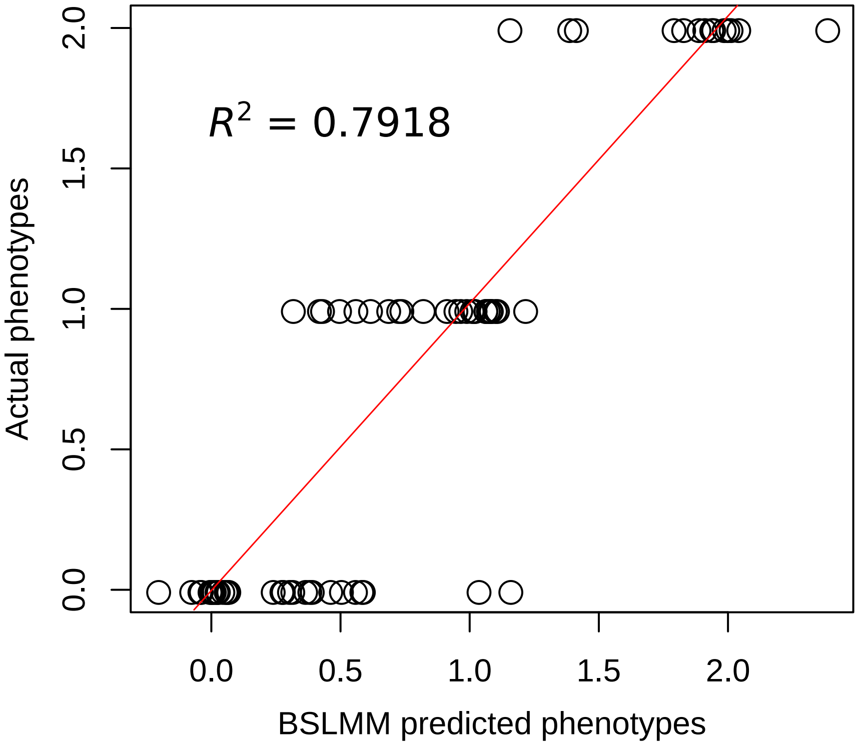

Supplementary Figure 5. Predicted Phenotypes.
Observed phenotypes versus phenotypes predicted by GEMMA Bayesian sparse linear mixed model. Source data are provided as Source Data file.

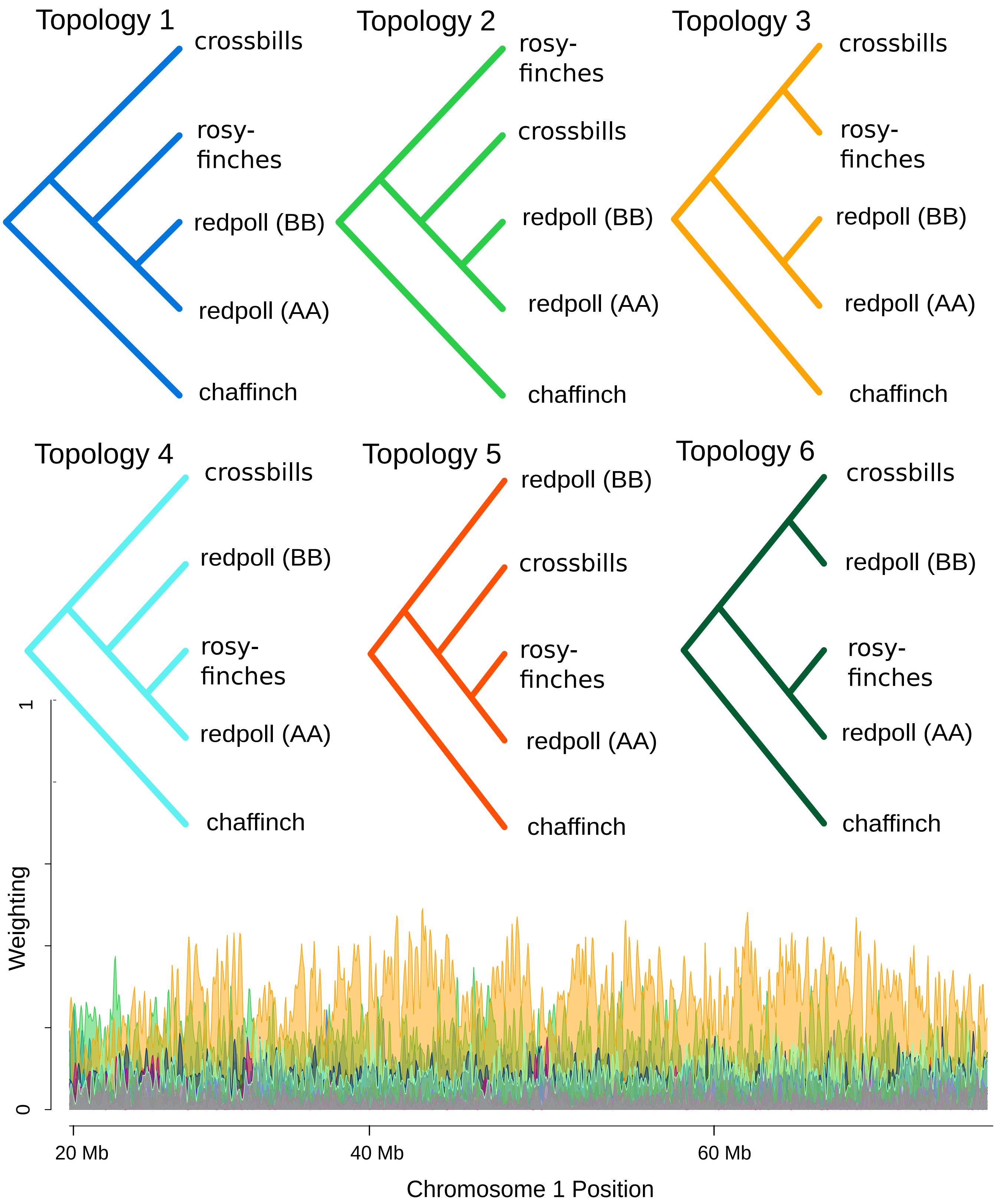

Supplementary Figure 6. Topology weighting using Twisst. Six of the fifteen total topologies tested and their weights for 100 SNP windows across the chromosome 1 inversion region. The most heavily weighted tree (Topology 3) indicates a sister relationships between the two redpoll inversion region haplotypes.

Supplementary Table 1. Simulation Results

Model 1 Simulations: Spatial competition

Mating (std. dev.)	Selection	Iterations Balanced	Average Karyotype ratios (AA:AB)*
0.5	Low	50	500:1
0.5	Medium Low	50	500:1
0.5	Medium High	50	500:1
0.5	High	50	500:1
0.75	Low	5	3:1
0.75	Medium Low	50	4:1
0.75	Medium High	50	5:1
0.75	High	50	7:1
0.95	Low	0	-
0.95	Medium Low	0	-
0.95	Medium High	0	-
0.95	High	0	-

Model 2 Simulations: Two populations

Migration	Selection	Iterations Balanced	Average Karyotype ratios
0.001	Low	50	24:1
0.001	Medium Low	50	50:1
0.001	Medium High	50	90:1
0.001	High	50	99:1
0.005	Low	50	5:1
0.005	Medium Low	50	10:1
0.005	Medium High	50	14:1
0.005	High	50	19:1
0.01	Low	50	2:1
0.01	Medium Low	50	5:1
0.01	Medium High	50	7:1
0.01	High	50	10:1
0.05	Low	0	-
0.05	Medium Low	0	-
0.05	Medium High	0	-
0.05	High	50	2:1

*BB karyotypes were equal to AA when averaged across all iterations
