# Supplementary material for: A supergene underlies linked variation in color and morphology in a Holarctic songbird
Source: Nat Commun. 2021 Nov 25;12:6833. doi: 10.1038/s41467-021-27173-z (PMC8616904; doi:10.1038/s41467-021-27173-z)
Supplement: Supplementary file 3 — Description of Additional Supplementary Files [file 41467_2021_27173_MOESM3_ESM.docx]

Description of Additional Supplementary Files

Title: Supplementary Data 1

Description: Sample list of redpolls

Title: Supplementary Data 2

Description: Extended list of genes associated with redpoll phenotype
