# Supplementary material for: A supergene underlies linked variation in color and morphology in a Holarctic songbird
Source: Nat Commun. 2021 Nov 25;12:6833. doi: 10.1038/s41467-021-27173-z (PMC8616904; doi:10.1038/s41467-021-27173-z)
Supplement: Supplementary file 6 — Reporting Summary [file 41467_2021_27173_MOESM6_ESM.pdf]

nature research

Corresponding author(s): Erik R Funk

Last updated by author(s): Oct 18, 2021

Reporting Summary

Nature Research wishes to improve the reproducibility of the work that we publish. This form provides structure for consistency and transparency in reporting. For further information on Nature Research policies, see Authors & Referees and the Editorial Policy Checklist.

Statistics

For all statistical analyses, confirm that the following items are present in the figure legend, table legend, main text, or Methods section.

n/a	Confirmed	
☐	☒	The exact sample size (n) for each experimental group/condition, given as a discrete number and unit of measurement
☐	☒	A statement on whether measurements were taken from distinct samples or whether the same sample was measured repeatedly
☐	☒	The statistical test(s) used AND whether they are one- or two-sided<br>*Only common tests should be described solely by name; describe more complex techniques in the Methods section.*
☐	☒	A description of all covariates tested
☐	☒	A description of any assumptions or corrections, such as tests of normality and adjustment for multiple comparisons
☐	☒	A full description of the statistical parameters including central tendency (e.g. means) or other basic estimates (e.g. regression coefficient) AND variation (e.g. standard deviation) or associated estimates of uncertainty (e.g. confidence intervals)
☐	☒	For null hypothesis testing, the test statistic (e.g. F, t, r) with confidence intervals, effect sizes, degrees of freedom and P value noted<br>*Give P values as exact values whenever suitable.*
☐	☒	For Bayesian analysis, information on the choice of priors and Markov chain Monte Carlo settings
☐	☒	For hierarchical and complex designs, identification of the appropriate level for tests and full reporting of outcomes
☒	☐	Estimates of effect sizes (e.g. Cohen's d, Pearson's r), indicating how they were calculated

Our web collection on statistics for biologists contains articles on many of the points above.

Software and code

Policy information about availability of computer code

Data collection	We as authors applied no commercial, open source, or custom code in the collection of our data.

Data analysis

Data were analyzed using a combination of open source and custom code, including the following programs for bioinformatic processing:
Trimmomatic PE v0.39
bwa v0.7.17
bcftools v1.10.2
NGMLR v0.2.7
SVIM v1.4.2

And additional analyses using:
SNPRelate v1.19.3 (R package)
conStruct v1.0.4 (R package)
adegenet v2.1.3 (R package)
plink v1.9
GEMMA v0.98.1
vcftools 0.1.16
Panther v16
SNPeff v4.3
SLiM v3.3

custom scripts from:
https://github.com/simonhmartin
https://github.com/erikrfunk

For manuscripts utilizing custom algorithms or software that are central to the research but not yet described in published literature, software must be made available to editors/reviewers. We strongly encourage code deposition in a community repository (e.g. GitHub). See the Nature Research guidelines for submitting code & software for further information.

Data

Policy information about availability of data

All manuscripts must include a data availability statement. This statement should provide the following information, where applicable:

- Accession codes, unique identifiers, or web links for publicly available datasets
- A list of figures that have associated raw data
- A description of any restrictions on data availability

Data are available at:
SRA (sequence data) - PRJNA753137
Dryad (vcf files) - https://doi.org/10.5061/dryad.q83bk3jjm

Field-specific reporting

Please select the one below that is the best fit for your research. If you are not sure, read the appropriate sections before making your selection.

☐ Life sciences ☐ Behavioural & social sciences ☒ Ecological, evolutionary & environmental sciences

For a reference copy of the document with all sections, see nature.com/documents/nr-reporting-summary-flat.pdf

Ecological, evolutionary & environmental sciences study design

All studies must disclose on these points even when the disclosure is negative.

Study description

Our study was aimed at uncovering the genetic basis of morphological variation in three closely related, and debated, species of redpoll finches (see below for species). We sequenced whole genomes from 73 individuals across all three species. In this study, we were interested in the evolutionary history of these taxa, the underlying genes and genetic architecture of phenotype, and attempting to discern the evolutionary mechanisms that are responsible for the maintenance and distribution of phenotypes in this species complex.

Research sample

Research Sample: Our samples included DNA extracted from blood and tissue samples from three species of finch (Acanthis flammea, Acanthis hornemanni, Acanthis cabaret). Rationale: These samples represent all three currently described "redpoll" species from across their range, and allow for comparisons of population differentiation, genetic divergence, and speciation. Redpolls as a group was chosen as these taxa are known to vary phenotypically, but vary little genetically, making the detection of phenotype related genes plausible. Representation: These samples were collected in an effort to represent all parts of the species distributions, and build upon previously collected samples from Mason and Taylor 2015. Mol. Ecol.

Sampling strategy

Sampling was haphazard based on species distribution, and sample availability

Data collection

DNA was extracted and sequenced using both short (Illumina) and long read (Oxford Nanopore) sequencing technology from blood and tissue samples of wild caught birds. Samples were obtained from museum collections. All museum voucher numbers are provided as supplementary data. No new samples were collected specifically for this study. DNA extractions were carried out by author ERF. Tissue samples were finely chopped using 200 proof ethanol and flame sterilized forceps, razor blades, and surfaces, and

	placed into sterile 1.5ml microcentrifuge tubes. Samples were lysed in a homogenizing solution of SDS buffer and protinase K on a heat block at 56 degrees Celsius. DNAs were precipitated out of an ethanol solution using salt in a microcentrifuge running at 13,000 rpm. All solutions were handled on a sterile lab bench using a standard set of microliter pipettes. All library preparations and sequencing runs were carried out by outside groups including the University of Colorado Denver Anschutz medical facility, and Colorado State University for short and long read data respectively.
Timing and spatial scale	Samples localities span multiple countries across the entire Holarctic, and were collected at all times of the year. We targeted at least 20 individuals from each of the three taxa to ensure we adequately captured genetic variation. We included samples from across their entire distribution to ensure as many sub populations were included as financially possible. Sampling timing and location was in part determined by the availability of samples in museum collections.
Data exclusions	One individual was dropped from our analyses due to sibling status with another sample
Reproducibility	All methods used were documented including software names, version numbers, commands, and arguments. Custom scripts were cited in text and can be found on github at:<br>https://github.com/simonhmartin<br>https://github.com/erikrfunk
Randomization	This study did not include experimental manipulations, or procedures involving treatment and control groups, and therefore did not include any randomization practices.
Blinding	This study did not include outside participants, or data acquisition stages that presented the possibility of bias based on the knowledge of sample information. Therefore no blinding measures were taken in this study.

Did the study involve field work? ☐ Yes ☒ No

Reporting for specific materials, systems and methods

We require information from authors about some types of materials, experimental systems and methods used in many studies. Here, indicate whether each material, system or method listed is relevant to your study. If you are not sure if a list item applies to your research, read the appropriate section before selecting a response.

Materials & experimental systems

n/a	Involved in the study	
☒	☐	Antibodies
☒	☐	Eukaryotic cell lines
☒	☐	Palaeontology
☐	☒	Animals and other organisms
☒	☐	Human research participants
☒	☐	Clinical data

Methods

n/a	Involved in the study	
☒	☐	ChIP-seq
☒	☐	Flow cytometry
☒	☐	MRI-based neuroimaging

Animals and other organisms

Policy information about studies involving animals; ARRIVE guidelines recommended for reporting animal research

Laboratory animals	No laboratory animals were used in this study.
Wild animals	DNA was sequenced from blood and tissues samples of museum specimens of previously wild caught birds, including three species of finch (Acanthis flammea, Acanthis hornemanni, Acanthis cabaret). Samples included adult birds of both male and female sexes. Samples were obtained from museum collections, and shipped to the University of Colorado Boulder on dry ice. No field work, or direct interaction with wild animals was conducted for this study. Samples collected by coauthors for previous studies were captured using a combination of salvage, and mist net techniques. Mist netted birds were either released immediately on site after taking a blood sample, or euthanized using thoracic compression for cataloging in museum collection as part of separate and previous studies. Other samples were provided by museums not involved in this study.
Field-collected samples	Our interactions with these samples were through museum collections and did not perform field work ourselves.
Ethics oversight	Samples for this study were provided by museum collections. All handling and processing of the samples on our end was done with approved permits and in accordance with the University of Boulder IACUC, the University of Iceland, Reykavik Institution of Life and Environmental Science, the Czech Academy of Sciences Institute of Vertebrate Biology, the Greenland Home Rule Government, the Danish Polar Center, and the Cornell University IACUC.

Note that full information on the approval of the study protocol must also be provided in the manuscript.
